# The loneliness of the long-distance ethnobotanist: a constructive critique of methods used in an ethnoveterinary study in Mongolia

**DOI:** 10.1186/s13002-021-00492-7

**Published:** 2021-11-17

**Authors:** Barbara C. Seele, Léanne Dreyer, Karen J. Esler, Anthony B. Cunningham

**Affiliations:** 1 Conservation Ecology and Entomology, Stellenbosch University, Private Bag X1, Matieland, 7602 South Africa; 2 Department of Botany and Zoology, Stellenbosch University, Private Bag X1, Matieland, 7602 South Africa; 3 School of Life Sciences, University of KwaZulu-Natal, King Edward Avenue, Pietermaritzburg, 3209 South Africa

**Keywords:** Ethnobotanical methods, Pastoralists, Fieldwork, Ethnobiology, Researcher well-being, Traditional ecological knowledge (TEK)

## Abstract

**Background:**

Fieldwork plays an important role in research projects across a variety of fields, especially in the multidisciplinary setting of natural and social science research. As is the nature of fieldwork, things do not always work out as planned, and yet this is not often written about. In response to the need for honest and transparent accounts of fieldwork, the purpose of this article is to review the methods used during fieldwork for the first author’s dissertation research on ethnoveterinary knowledge.

**Methods:**

To critically review and reflect on the fieldwork methods used for an ethnoveterinary study in Mongolia, we compare the theory underpinning each method with the practical reality of implementing the method in the field. From this comparison, we draw out and discuss a number of key themes.

**Results:**

Eighteen methods and approaches used for the research project are reviewed and compared. From this, we distil and further discuss the following five overarching themes: reflections on specific data collection methods (free listing, semi-structured interviews with interpreters, voucher specimen collection); assumptions around involving local people; power dynamics; gender relations; and researcher well-being.

**Conclusion:**

By juxtaposing the theory and practical reality of the methods used, we highlight many potential fieldwork challenges and, within this context, offer general pointers, especially for novice female researchers doing fieldwork in foreign countries. A critical review of this type, where the experience and use of various methods, techniques, and approaches are openly shared and evaluated, is a contribution to selecting, adapting, and fine-tuning the methods best suited to a particular research context.

## Background

The incorporation of traditional or indigenous knowledge in scientific research has received growing interest over the past few decades [1–3]. Although the importance of local knowledge is increasingly recognized, for example in solving environmental problems [4], there are concerns that some complex social-scientific research issues have not received the attention they deserve [5, 6]. Among others, these issues include understanding and respecting the context in which traditional knowledge is situated [7], sensitivity to local concerns, and the quantitative analysis and reporting of data that have been collected through social interactions [6, 8]. Within the context of an ethnobotanical research dissertation, a review of the selected fieldwork methods offers a window into the complexities and nuances of ethnobotanical fieldwork.

Consisting of both social and scientific research, dissertation fieldwork focused on exploring and understanding the ethnoveterinary knowledge and practices of Mongolian herders. Ethnoveterinary knowledge describes local or traditional knowledge regarding livestock health [9]. As this knowledge includes the practices, beliefs, and relationships of humans with other living beings (for example, herders and their livestock) and the environment, it can be described as a form of traditional ecological knowledge (TEK) [10]. In addition, because ethnoveterinary knowledge and practices largely focus on medicinal plant use, ethnoveterinary research primarily uses ethnobotanical research methods.

Based primarily on ethnobotanical methods, fieldwork for the dissertation took place in Mongolia during the summer months of 2014 and 2015, with the aim of sensitively investigating ethnobotanical, specifically ethnoveterinary, knowledge and practices of nomadic Mongolian pastoralists. Fieldwork included semi-structured interviews, held through an interpreter, on the medicinal plants used for livestock, and consisted of weeks on the steppes travelling between herding families, using a mixed-methods data collection approach.

Several books, manuals, and journal articles have been published on ethnobotanical research methods [11–18], including ethnoveterinary research techniques [19]. Although these texts are important for explaining general methods and approaches, the specific cultural and political contexts in which these methods are used can differ significantly. In addition, the interaction of multiple factors of the individual researcher with the research context adds complexity. Examples of such factors include the identity, gender, age, country of origin, as well as the general and psychological well-being of the researcher. Choosing, applying, and adapting the most suitable research and fieldwork methods for a specific research context can be challenging. Furthermore, many unexpected problems may arise such as negotiating with figures or institutions of authority and dealing with research fatigue [20]. These “skeletons in the methods cupboard” are rarely examined, even though some of the most significant lessons arise from reflecting on and better understanding why problems occur in field research [21, 22]. The practical challenges that long-distance researchers face often differ according to the context, but are seldom written about. Droughts happen, families move, sheep need shearing, policies change, and other day-to-day complexities, often unexpected and unrelated to research, can play a large role in shaping research outcomes [23].

Since the development of the 1992 Convention on Biological Diversity (CBD) [24] and the adopted 2010 Nagoya Protocol [25], the general ethnobotanical research approach is to develop collaborative, equal partnerships with local research partners [26] and ensure the “fair and equitable sharing of benefits derived from the utilization of genetic resources” (Article 1, Nagoya protocol) [25]. This requires “prior informed consent” before gaining access to traditional knowledge and emphasizes that the sharing of traditional knowledge is based on the consent of both parties to “mutually agreed terms” (Article 7, Nagoya Protocol) [25]. Although fieldwork for the research project was done in accordance with these agreements and the primary researcher had previous experience of the research area, as is often the case in fieldwork, unexpected and difficult challenges arose during the research period and the understanding gained from these experiences could hold lessons for other researchers.

Although good lessons come from failed experiments and problematic fieldwork, scientists rarely mention the research and personal difficulties experienced during data collection, if at all [27–29]. However, although still uncommon, deliberated reflections on methodologies and associated problems are slowly receiving some recognition; for example, a dedicated ‘setbacks and surprises’ section in* Restoration Ecology*, occassional editorials offered by the* Journal of Ethnobiology and Ethnomedicine* [30], and a discussion by Shackeroff and Campbell [6] on the complexities of using TEK for conservation research, with a specific focus on power and politicization, ethics, and situated knowledge. Further examples include Mandel’s [20] reflexive account of the differences between expectations and actual experiences regarding fieldwork in Benin, and Berlin and Berlin’s [16] account of third party intervention in establishing rapport and the informed consent process. In terms of ethnographic accounts, where the focus is on a complete description of a culture-sharing group [31], generally, little is written about reflections on fieldwork problems and possible reasons thereof. However, exceptions include Hewlett’s exceptional volume *The Secret Lives of Anthropologists: Lessons from the Field* [18] and Nigel Barley’s *The Innocent Anthropologist* [32]. Even less is written on certain important topics such as gender roles, bias, and associated vulnerabilities [33]. These are important topics, worthy of reflection and discussion.

Within a research project with the aim of identifying conservation implications of Mongolian herders’ ethnoveterinary knowledge [34], this paper offers a critical review of the methods and approaches used to collect and understand ethnoveterinary knowledge of nomadic Mongolian herding families as a foreign researcher. Rather than focusing solely on the success stories of our research project [34, 35], we take a theory versus practice approach to highlight and review some of the challenges faced. Our hope is that these reflections hold valuable lessons and offer support and relatability to other researchers in their fieldwork accounts and to offer some guidance to those planning fieldwork projects across similar fields.

## Methods

The lead author, from South Africa, completed a research-based MSc dissertation on the ethnoveterinary knowledge of Mongolian herders in 2017 [34]. The catalysts for this project came from her growing up on a working South African livestock farm and, then, participating in the Mongol Derby in 2013—a 1000 km self-navigated and self-sufficient endurance horse race, in which competitors ride Mongolian horses and overnight with pastoralist families. Partaking in such an endurance event, central to Mongolian society, represented an opportunity for experiential learning and familiarized the lead author with the Mongolian pastoralist way of life.

An initial fieldwork trip to Mongolia in autumn 2014, aimed to establish local research contacts, included participation in a Mongolian rangelands conference, piloting interview questions and techniques, and conducting preliminary fieldwork. Thereafter, the authors established collaborative arrangements between the first author’s South African university of registration and a Mongolian university. Notably, ethical clearance for the project was obtained separately from both institutions, with ethical guidelines as stipulated by the International Society of Ethnobiology [36] strictly followed at all times. The main data collection period then took place over an intensive three-month period during the summer of 2015. The research used a mixed method approach that included snowball sampling, free listing, the use of images in a reference book, and semi-structured interviews with herder families via a local interpreter. In addition, observation schedules and daily fieldwork journal entries were used to record fieldwork experiences.

Fifty interviews were conducted with Mongolian herder families in the north-central region of Mongolia. Throughout the fieldwork period, the researcher endeavored to learn about, respect, and follow culturally appropriate norms and behaviors. For example, upon meeting the families, introductions were made (similar to Sternberg’s [29] approach), tokens of appreciation were offered to the families (various hard-to-come by food items, as suggested by the interpreter, as well as small souvenirs from South Africa, e.g., key chains), followed by the sharing of tea or a meal, before interviews began. The background and reasons for the research were explained, and respondents were given a subject information sheet (in Mongolian) explaining the aims and objectives of the study, as well as contact details of the primary researcher. Interviews were only conducted if informed signed consent was given. Interviews were held in Mongolian through an interpreter, with responses to each section translated into English, which the primary researcher recorded using handwritten notes. Audio recordings (*n* = 35) and photographs were only used if respondent consent was given. As three different interpreters were used during the fieldwork period, audio recordings were transcribed and translated by an official translation company, with a signed confidentiality agreement, to account for possible interpreter bias.

Interviews began with a free listing opportunity during which herders were asked to list the medicinal plants that they used for their livestock. Position and frequency of mention [14, 37] allowed the research team to become familiarized with the locally important and useful categories and plants through an emic approach. After free listing, herders were given the opportunity to page through the reference book ‘*Flowers of Mongolia*’ [38] containing photographs of flowering plants with scientific species names. This was based on a method used and described by Thomas et al. [15]. Herders were asked to indicate which plants were used for ethnoveterinary purposes and to describe the local names, uses, dosage, collection, storage, preparation, and administration methods. Plants that were recognized and used for other purposes (for example, human medicinal) were also recorded and used as a proxy for general plant knowledge. While paging through the book, many respondents verified the cultural salience of plants mentioned during the free listing session.

Following from the reference book, participants were asked a number of open- and closed-ended questions designed to gather descriptive data and respondent demographic information (age, education, livestock numbers, migration patterns), livestock illnesses and disease, knowledge transfer and threats, and to examine herders’ perceptions of medicinal plant use, knowledge, and conservation. Herders were then given an opportunity to ask their own questions about the research. Where feasible, herder reports were verified by personal observations of livestock numbers, medicinal plant storage methods, and the location of nearest water sources. Ethnographic notes and journal entries were made at the end of each day.

Where possible, voucher specimens were either collected in the field together with respondents (ideally), with the knowledgeable horse-guide (an integral member of the research team), or from dried specimens that respondents had stored for winter. Pressed specimens were collected for later identification and storage at the herbarium of the National University of Mongolia’s Traditional Medical Institute. Fieldwork was initially conducted using a motor vehicle for transport between herding families, allowing for preliminary preparations to be made and for the research project to get underway in a safe manner. Thereafter all fieldwork was done on horseback, together with a local guide.

To gain an understanding of the importance and use of medicinal plants from the perspective of the medicinal plant trade, we developed specific interview schedules for market sellers that included questions about medicinal plants sold for livestock and human use.

**Fig. 1:**
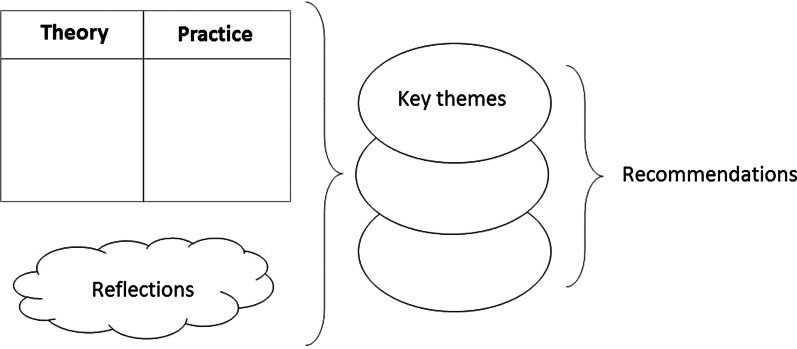
Structure used to critically review the methods used for an ethnoveterinary study in Mongolia

To critically review the various methods used for the described research project, we compare the theory and rationale behind each method or approach with the practical reality, experience, and outcome of applying the method. In addition, field-note entries and participant observation schedules were analyzed with prompts from ‘*Reflections on Research—The Realities of Doing Research in the Social Sciences*’ [39] to stimulate natural, free-flowing reflections on the fieldwork experience and problems encountered. From the reflexive process of juxtaposing theory and practice together with the principle researcher’s reflections, we draw out key themes for further discussion using relevant literature and, ultimately, develop corresponding recommendations (Fig. 1).

## Results

**Fig. 2:**
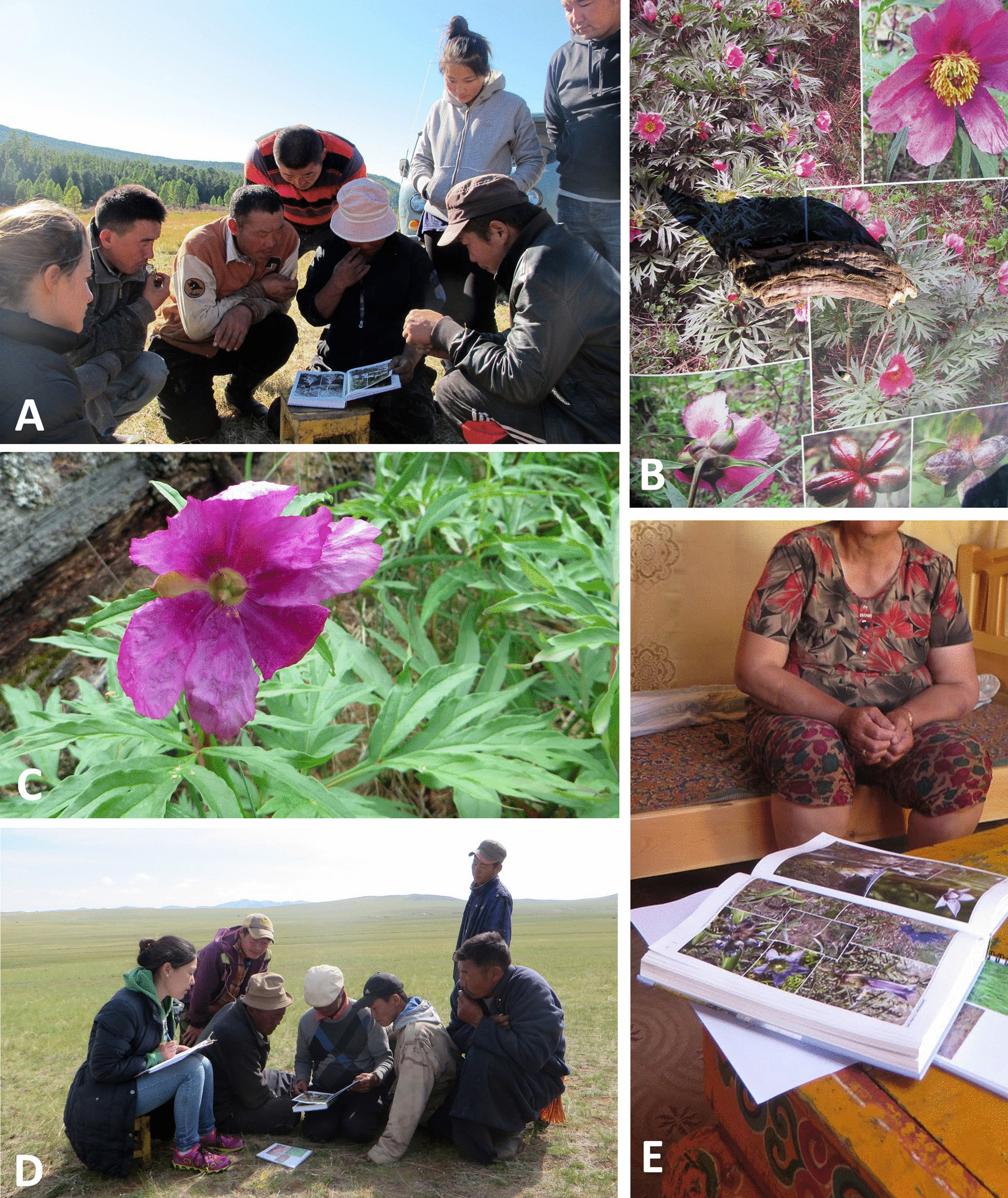
Interviews with Mongolian herders about ethnoveterinary medicinal plants with the use of a reference book *Flowers of Mongolia* (**A**). An example of an ethnoveterinary medicinal plant *Paeonia anomala*, with the corresponding reference book entry and dried root specimen (**B**), and the same plant found growing in the wild (**C**). Interviews with men were usually held outside (D), while those with women usually took place inside the *ger* (felt tent home) (**E**). Photograph credits: A, C–E: B. Seele, B: L. Dreyer

**Fig. 3:**
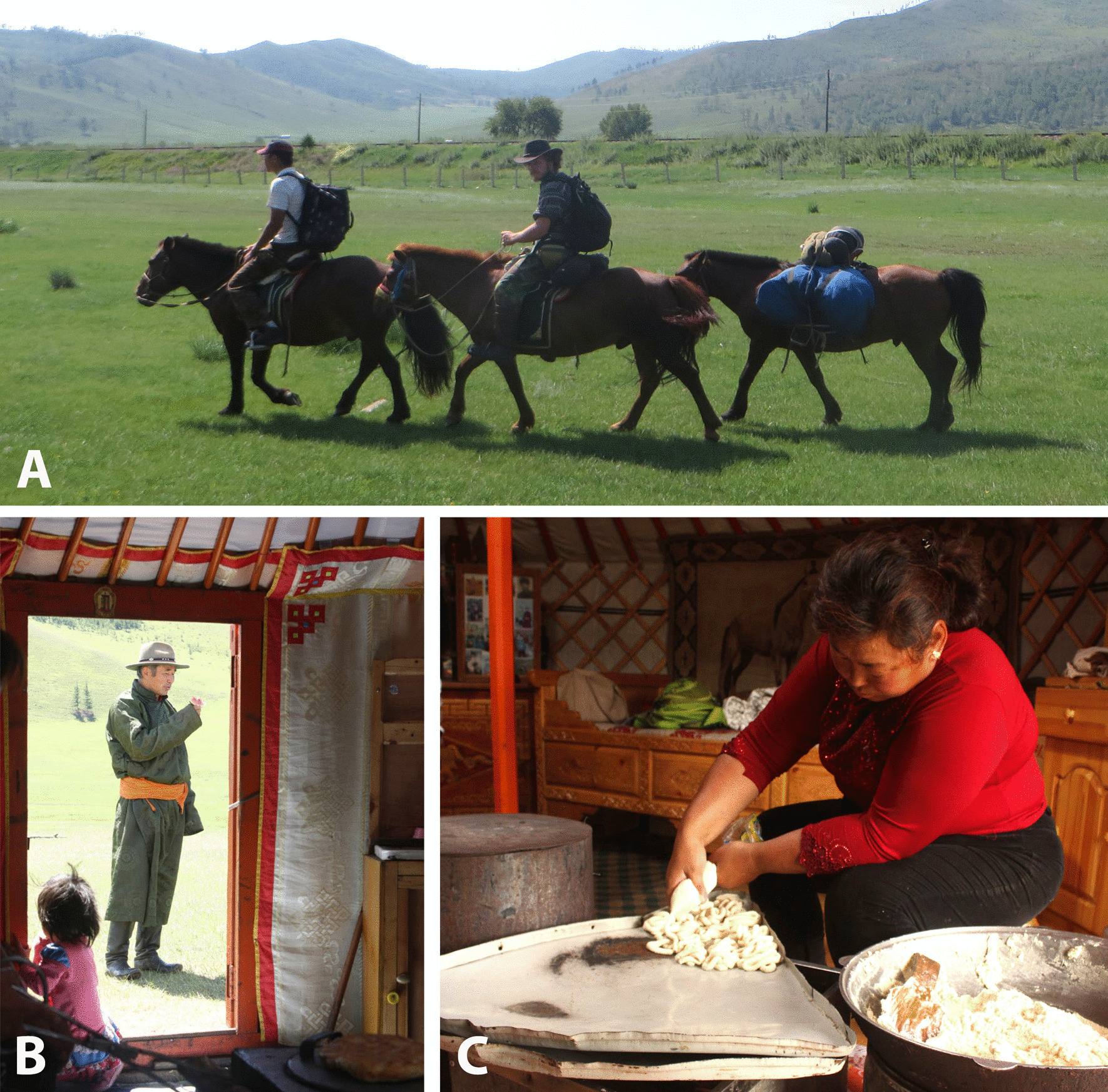
Various fieldwork approaches used for the collection of ethnoveterinary data. Fieldwork was conducted using horses as the main form of transport (**A**). Interviews began with introductions by the horse guide, a respected community member, (**B**) and were conducted in a manner that allowed female respondents to complete their daily chores such as the making of *aaruul* (dried curds) (**C**). Photograph credits: A: B. Seele, B, C: H. Wiese

Data collection for the research project on the ethnoveterinary knowledge of Mongolian herders [34] included several diverse fieldwork methods and approaches (Figs. 2, 3). A total of 18 methods were reviewed individually by comparing the theory behind each method with the outcome of using and applying the method (Table 1).

**Table 1:** Theory versus practice: a chronologically ordered review of methods used for ethnoveterinary research in Mongolia. Adapted from Seele et al. [34]

Methods used	Theoretical approach	Practical reality	Comments
Experiential learning, in this case, participation in the Mongol Derby (1000 km self-supported endurance horse race)	Better understanding of the context in which local knowledge is practiced [6]. Establish rapport with local knowledge holders [14]	Strength-related endurance activities such as long-distance horse riding and wrestling are central to Mongolian society. Participating in an endurance race allowed the first author to earn the respect of local herders. Riding Mongolian horses and overnighting with herder families allowed for insight into the Mongolian pastoralist way of life	Highly beneficial: increased understanding of the Mongolian pastoralist context. As a female researcher, participating in a long-distance horse race, as opposed to in other male-dominated endurance activities such as wrestling, allowed the first author to gain and earn the respect of local herders. Further, it enabled the establishment of important relationships with knowledge holders, interpreters, and local guides
Collaboration with local Mongolian university	Establish local research partners [24, 36]	Administrative benefits, including research visa and affiliation with local university. The use of the local Mongolian university herbarium was important for identification and storage of voucher specimens	Although more about administrative steps, this was a crucial part of the research process, and it is hoped will play a role in the distribution of findings in Mongolia
Ethical clearance and prior informed consent	Prior informed consent [25, 36]	Obtained from two universities; instrumental in explaining the intention and motivation behind the research	It proved invaluable to receive ethical clearance from a local institution as it increases research credibility and offers protection to both respondents and researchers
Employment of driver and interpreters	Local involvement in research team and research logistics [36]	Employing a local driver proved challenging due to unforeseen power and gender relations. The driver’s personal agenda influenced decisions around the selection of respondents and route. The driver became aggressive when questioned. Finding a good interpreter was difficult as they are in high demand and easily find other jobs. Interpreter bias became a problem in some interviews	The importance of a driver is often underrated, especially across a language barrier. Being from the local area does not automatically mean that research team members have the necessary skills, knowledge, and cultural sensitivities for research on indigenous knowledge [28]. Cultural sensitivity can also be misused
Recording of interviews	Use of hand-held audio recorder during interviews [14], only if consent is given	Seventy percent (*n* = 35) of interviews were recorded after consent was given. In retrospect, the manner in which the interpreter explained the recording may sometimes have influenced respondents' reaction toward the recording	Transcription and translation of recordings gave valuable insight into interpreter bias. It is important to be extremely sensitive with recordings [39]
Snowball sampling	A nonprobability sampling method, often used in field research, where interviewees are asked to suggest additional people for interviewing [40]	Mongolian pastoralists have an extensive social network, which was key to locating knowledge holders and establishing trust. Contacts from both the Mongol Derby and through the horse guide assisted with snowball sampling	Although this sampling method generally worked well, in one situation, suggestions from respondents were translated incorrectly by the interpreter due to the driver’s personal agenda (*n* = 1)
Free listing	Free listing can provide insight into culturally important plants and ailment categories [14]. As free lists are not exhaustive [37], where possible, inventories from free listing were supplemented and crosschecked using a plant reference book [15]	Free listing seemed to allow respondents to become comfortable with the interview situation and encouraged a more balanced positionality of power between researcher and respondent, enabling the interviewee to become the teacher and the researcher to become the learner	Using position of mention and frequency of mention enabled the research team to become familiarized with locally important and useful plants. Free listing also allowed for the quantitative analysis of plant use and importance via species accumulation curves, use-value, informant consensus factor, and fidelity level [34]
The use of photographs in the reference book *Flowers of Mongolia* [38] for ethnoveterinary medicinal plant inventories	Interviews held ex situ with plant photographs as a reference tool [15]	An adult (65+ years) literacy rate of 97.3% in Mongolia (2018) [41] substantiated the use of the reference book method. In general, herders reacted positively and with much interest to the book. However, four respondents mentioned having poor eyesight and chose not to use the reference book	This ethnobotanical data collection method can be used effectively in situations where respondents feel comfortable with seeing depictions of plants in a two-dimensional format, and where respondents do not have eyesight difficulties. Notably, there may be situations where hearing impairments also need to be taken into account
Voucher specimens	Good quality herbarium specimens are crucial to ethnobotanical (and ethnoveterinary) studies [11, 13, 14]. Researchers should consider both conservation-related and local cultural concerns [13]	In general, voucher specimens were difficult to collect for all mentioned plant species owing to an ongoing drought at the time [42], herders being busy, the distance to medicinal plant locations, and cultural objections	Concerns were raised about the use of a GPS to record voucher specimen locations, perhaps due to fears around mining-related activities and bioprospecting
Use of a compass, maps, and GPS for navigation	Used to determine geographical distance for planning routes and to record interview and voucher specimen locations	Not everyone uses the “western” approach to map reading and direction. Locally, time and distance measures were done taking horseback travel, local relief, and the availability of jeep tracks into consideration. Compass directions were also interpreted in different ways	Researchers need to be flexible in terms of when and how to get to a specific area, and should prepare for the possibility of cultural differences in map reading and navigation
Interviews	Use of semi-structured interview with open- and closed-ended questions for the collection of ethnobotanical data [14]	Conducting good interviews in terms of scientific and social quality was sometimes challenging, especially when non-interview issues came up during the interview. Questions regarding herd size and demographic information made some respondents feel uncomfortable and were then omitted	Other researchers, for example Heffernan et al. [43], also recorded that certain questions were uncomfortable for respondents. Researchers should receive specific training in conducting interviews from experts and practitioners within the social sciences, such as anthropologists
Travelling the local way (in this case on horseback)	Establish rapport with local knowledge holders and participant observation [11, 14]. Reduce imbalance in position of power [6]	Although travelling on horseback meant a decrease in the daily distance covered, it soon became clear that this was the more appropriate mode of transport. Respondents seemed to feel more at ease, with the common topic of horses establishing some connection and leading to naturally stimulated conversation around livestock care and valuable insight into life on the steppes	Travelling on horseback positively affected project success by enabling the research team to place ethnoveterinary knowledge in the context of the Mongolian herding way of life. In addition, being passionate about horses, travelling on horses-back, and being around horses had a positive influence on the primary researcher’s motivation levels during the project and added to her overall well-being
Employment of local horse guide on research team	Understand the context of traditional ecological knowledge [6]. Local involvement in research team and research logistics [36]. Establish rapport with local knowledge holders [14]	The horse guide was a well-known and respected community member. He suggested which families to visit, and facilitated introductions of the research team and project. Additionally, he assisted with the collection of voucher specimens	The assistance of the horse guide with horse-care, logistics, and introductions to knowledge holders was invaluable during fieldwork
Having a partner/good friend as field assistant	Researcher health and well-being is critical for the success of a research project [44, 45]. Importance of fitting in with social norms, i.e., having a husband	The assistance and presence of a partner during the often strenuous and lonely fieldwork proved crucial in terms of emotional and practical support. During fieldwork, the primary researcher introduced her partner (field assistant) as her husband, which prevented any untoward responses, increased her status in a patrilineal society, and reduced vulnerability associated with doing fieldwork as a foreigner	Researcher well-being and gender issues around vulnerability are important to consider before, during, and after fieldwork [33, 46]
Use of observation schedules and a fieldwork journal	Suggested use of daily entries into a fieldwork journal and observation schedules for each interview as well as other noteworthy experiences [14]	Observation schedules allowed for the recording of finer, often crucial, details that are easily forgotten or overlooked in the full and demanding schedule of fieldwork. In addition, time spent on journaling offered an opportunity for debriefing, processing, and reflecting on fieldwork	Keeping a journal and recording observations proved invaluable during the later analysis of interviews to understand the context of the interview situation and fieldwork in general. However, it was sometimes challenging to add qualitative data to quantitative analyses of medicinal plant use in a meaningful and structured way
Market surveys	Interviews were conducted with market sellers at two major markets following guideline described by Martin [14] and Cunningham [13]	Market sellers reacted with suspicion to questions relating to plant sales. A relationship was established with only one market seller	More time was needed to establish relationships with market sellers before conducting interviews. The ca. 60 years of being a Soviet satellite state could be a factor in mistrust and guardedness relating to interviews and being questioned
Assistance from boundary organizations	Boundary organizations can be instrumental in bridging the gap between research and practice, especially if they have experience and are familiar with the local context [47, 48]	Staff from the Deutsche Gesellschaft für Internationale Zusammenarbeit (GIZ) (Biodiversity and Adaptation of Key Forest Ecosystems to Climate Change Programme) offered invaluable support with interpreters, sourcing horses, and fieldwork logistics	It is crucial to identify and receive assistance from supportive boundary organizations. This can take time and should be factored into research planning. More emphasis should be placed on learning from and working with these organizations
General support	There is a need for new researchers to have support systems in place during the often-times stressful and lonely fieldwork, as well as post-fieldwork for debriefing and reflection [39, 44]. Researcher fatigue can result in inflexibility, impatience, and an inability to put aside personal bias, making fieldwork very challenging [20]	Academic and motivational support was provided by university supervisors during the course of the project and fieldwork. Professional psychological support was sought after fieldwork had been completed for post-traumatic stress and played a critical role in completing the research project and dissertation	Importance of psychological support during and after fieldwork to prevent researcher exhaustion and “burn-out”

## Discussion of key themes and related recommendations

Although personal experiences, anecdotes, and reflections are not often described or emphasized in ethnobotanical literature [30], the importance of honest, transparent accounts of fieldwork experiences is becoming increasingly apparent. For example, the memoir essays of ethnobiologists and anthropologists describing first experiences in the field [32, 49, 50], recollections of failed fieldwork [51], and a description of “lost” days in the field [20] offer a wealth of real-world guidance and a human-perspective to students and researchers.

By critically reviewing the (largely ethnobotanical) methods and approaches used for our ethnoveterinary study in Mongolia and focusing on *how* we collected data rather than *what* we collected [52], a number of key themes emerged that we discuss in more depth. These overarching themes include reflections on the appropriateness, success, or limitations of chosen methods and related recommendations. Notably, these reflections are from personal experience and a specific context, but we hope are useful to both new and experienced researchers in similar fields.

### Reflecting on medicinal plant data collection

#### Free listing

Reflecting on the interviews held with herder families, the importance of sequence and position of power becomes apparent. Beginning the interview process with a free listing opportunity allowed respondents to feel more comfortable with the interview situation and to encourage a more balanced position of power between the research team and respondents. Recording position and frequency of mention enables insight into local plant use and importance [37], as well as local nomenclature and awareness of possible cultural sensitivities. The subsequent use of the reference book as described by Thomas et al. [15] enabled herders to then verify free listed plants with photographs from the reference book.

#### Reference book for photograph identification

The use of a reference book to collect medicinal plant data was appropriate for Mongolian herders, as the country has a 97.3% adult literacy rate in 2018 for men and women 65 years and older [41]. This was confirmed in our interviews, where 98% of respondents (*n* = 50) had received formal schooling. In practice, the reference book was met with much interest from the herders and allowed herders to direct the interview and share knowledge with family members and the research team. Although the reference book only represents approximately 15% of vascular plants in Mongolia, it has large and detailed color photographs, making it well-suited as an ethnobotanical reference tool during interviews. However, the use of a reference book during interviews also presented some limitations. Four respondents mentioned having poor eyesight and could not see the plant photographs in the book clearly. This must be considered when deciding to use a reference book, especially where knowledge is held by elders of the community. A recommendation for those using visual methods with elders in very impoverished or rural areas would be to carry reading glasses of a few different strengths (these would also be excellent gifts for participants). Furthermore, as the book displayed photographs of two species (same genus) per page, respondents sometimes indicated, by vaguely pointing at all photographs on the page, that both species were used for a particular ailment, but both voucher specimens could not always be collected. To navigate this, we decided to analyze species use and importance using ethnospecies related to local folk names recorded from interviews as a unit of analyses, rather than botanical species. This method is described by Hanazaki et al. [53] and also used in an ethnobotanical study in the Bolivian Amazon by Reyes-García et al. [54].

#### Semi-structured questionnaire

The final part of the interview process consisted of a semi-structured questionnaire with open- and closed-ended questions. When informants felt uncomfortable with answering specific questions during the interview process, we omitted the questions. Examples included questions on livestock numbers, which reflect financial wealth and status. In retrospect, it would have been valuable to (re)explain and remind the interpreter of the reasoning behind these questions and, if feasible, to seek alternative ways of phrasing the questions. Adjusting questions to get at the same type of data (or a proxy) can make the interview participant more comfortable and, therefore, also the interpreter. Consulting with an interpreter/assistant to explain why a specific question is problematic, and asking their advice on alternative question can help a researcher find a culturally or personally less sensitive option. For example, rather than asking for herd numbers, we could have asked whether participants felt that their herd size was on the smaller or larger side for their community. Establishing rapport and negotiating translation terms with research assistants and interpreters are important for such troubleshooting [55]. As Hallowell et al. [39] describe, the trajectory and outcome of an interview cannot be predicted, including possible reactions or emotions that specific situations or questions will elicit in others. Reviewing the interview schedule item-by-item to find potential sticky areas for the interviewer and doing pilot surveys play an important role in problem-prevention; but, ultimately there is no such thing as a safe question and continuing with the interview may involve making some very challenging, spontaneous decisions. Researchers can support themselves by preparing for the unexpected, gaining interview skills, and allowing time to practice conducting interviews with sensitivity, diplomacy, intuition, and skill.

Audio recordings and photographs were only taken if prior consent was given by respondents. That not all interviews were recorded indicates that participants felt able to make a decision around these options and indicates a positive, responsive outcome rather than an incomplete one. Although not experienced directly, difficulties and misunderstandings around the use of audio recorders (or video or photographs) can easily arise in interview situations, as described by Hallowell et al. [39], and researchers should be prepared for possible cultural and personal sensitivities around recording interviews. This can be done through anthropological training in the art and science of conducting interviews and how to deal with non-interview-related issues that may arise during an interview. Anthropologists place emphasis on research objectives being explained and understood locally and that “residents find in their participation a value that transcends whatever immediate remuneration we offer” [56].

#### Voucher specimens

After interviews, voucher specimens were collected together with respondents (where possible), under the guidance of the knowledgeable horse guide, or from dried specimens stored by respondents for later use in winter and spring. In general, voucher specimens were difficult to collect for a number of reasons. Specimen collection was hampered by the ongoing drought at the time of fieldwork [42], resulting in many medicinal plants not growing in their usual locations. Concerns around locating water sources and the many activities around sheep shearing season meant that many herders were too busy to show the research team examples of medicinal plants in the field, especially where plants grew further away or in difficult to access regions. Furthermore, many medicinal plants were described as growing in sacred mountain or forest areas, far away from the *ger* (felt tent home) and were only visited when out herding and sometimes only accessible for men. The research team also experienced cultural objections to the collection of voucher specimens, with one respondent requesting that no plants be collected as collection would result in the development of a thunderstorm. In addition, some respondents had fears around the use of a GPS and knowledge of the location of certain plants being misused, and therefore, accordingly, the research team decided to tread lightly in terms of voucher specimen collection.

Collection of voucher specimens is necessary, especially in biodiversity-rich developing countries [57], such as Mongolia, which contains two World Wildlife Fund (WWF) Global Ecoregions [58]. Although Cunningham [13] mentions sensitivity to cultural concerns as one of five best practices for collecting plant specimens, satisfying academic requirements of voucher specimens, while taking cultural fears and sensitivities into consideration, can be difficult to navigate. In retrospect, possible ways to allay fears around specimen collection could be taking the time to explain the reasons for collection voucher specimens, perhaps showing photographs of a local herbarium, and possibly making labelled herbarium specimens together with respondents as a tool for sharing local ethnoveterinary knowledge. Furthermore, we might have modified the voucher collection, making GPS measurements less obtrusive. Collectors should be free to make adjustments by considering the purpose of each data point. In retrospect, getting measures outside of homesteads or omitting GPS for vouchers, instead finding approximate altitude and geographic positions from a map, would suffice to indicate a plant’s growing zone.

#### The essence of time

During our fieldwork, it soon became apparent that herders and their families are generally very busy with daily and seasonal chores, and only have limited time for interviews and lengthy discussions, if at all. Having free listing, the use of a reference book, and a questionnaire as part of the interview process was time consuming. Several herders seemed tired after the initial free listing and were not interested in looking at the reference book, or simply paged through very quickly. Of the 50 respondents interviewed, only 30 (60%) completed all three sections of the interview due to time constraints, poor eyesight, and interview tiredness. While researchers cannot control local respondents’ mental or physical conditions, they can manage time spent on interviews. While an interviewer has a respondent sitting right with them, it is a hard decision to end an interview, saving some of it for later. However, in this case, with 40% of interviews not being completed, it may have been better, in retrospect, to break the interviews into separate sessions. For example, we may have conducted the free lists with families on the way out from our starting point, then collected the reference book plant-recognition data and vouchers on the way home. This would have added no total time to data collection but have the advantages of breaking the time up for participants and adding to rapport on the return interview.

During interviews, we noticed that some participants seemed reluctant to share local knowledge for various reasons including having more pressing concerns as well as cultural or linguistic difficulties. The hesitancy to share local knowledge has also been recorded by Luseba and Tshisikhawe [59] and Sternberg [29], and reiterates the importance of having enough time to establish good rapport with respondents, local interpreters, and research team members, and to form relationships based on trust and understanding. This speaks to the potential need to shift from focusing on the quantity of interviews to rather focusing on the quality of interviews, even if this means less data and more time.

Although we had planned to gather medicinal plant market data, the reality was quite different from what we expected and we learned some important lessons. By the time we had conducted interviews with four medicinal plant sellers at two of the biggest markets in Mongolia, drawn maps of the markets, and established a significant relationship with one seller, it became clear that much more time was needed for further and more in-depth market-related interviews as most sellers, understandably, seemed wary and suspicious of a questioning foreigner. To build trusting relationships with medicinal plant collectors and market sellers, and to understand both the individual and social contexts, requires insight, understanding, and a large amount of time.

Although gaining an understanding of the specific context in which fieldwork takes place is key to better understanding, analyzing, and reporting data [20], the time required to build relationships and form a multidisciplinary research team with the correct local people is not always available. In light of this, funding bodies and educational institutions need to understand that not only finances, but also time, together with passion and purpose are essential resources for research of this nature, especially for fieldwork where both geographical and cultural loneliness are very real challenges.

### Assumptions around involving local people

In our fieldwork, although the majority of interviews were harmonious, with herders enthusiastic about sharing their knowledge, unfortunately, a number of interview situations deteriorated, with herders walking out, seemingly angry, and the interpreter visibly upset. Due to the language barrier, this situation was very difficult for the primary researcher to navigate and interpret, and resulted in feelings of being side-lined and out of control together with a loss of confidence and motivation. Ultimately, the translation and transcription of audio recordings retrospectively assisted in better understanding the influence of the interpreter’s personal agenda on interviews and respondents’ reactions, as well as translator bias. From the recordings, we realized that one interpreter sometimes mistranslated respondents’ answers to shorten an interview. Another interpreter used the interview situation to share her religious views, which upset some of the respondents, leading to a very heated and uncomfortable situation with a breakdown in communication, and, ultimately, the interview having to be cut short. This could be why very few spiritual/religious plant uses or practices were mentioned during the interviews. In retrospect, it is important to set aside time for a debriefing after each interview or at the end of the day, to process challenging topics or questions and to offer interpreters an opportunity to expand on participants’ responses and perceptions [55]. Furthermore, sometimes, rather than finding a new interpreter, sensitizing the interpreter to potential translator bias before the start of fieldwork can assist. Even if an interpreter or field assistant comes well-recommended or has previous research experience, they may require further training, specific to the project’s needs. What seems obvious to a researcher (not to ask leading questions, not to opine on respondents answers) is not necessarily obvious to the assistant. For example, a researcher needs to explain that the interpreter cannot show how they feel. In our case, training the interpreter in terms of remaining objective and not judging an interviewee’s responses even if these go against personal beliefs/religious perspectives could have helped with interpreter bias. In addition, although difficult, having the entire research team fluent in Mongolian would have been ideal, if not unfeasible.

Further uncomfortable problems arose when the primary researcher’s intention to be culturally sensitive was somewhat misused by a driver on the research team. On one occasion, the driver gave incorrect route information to shorten the fieldtrip and, on another, convinced the interpreter to incorrectly translate an interviewee’s response regarding other possible interviewees in the area (snowball sampling). Commitments made to the research project (time or employment) by local team members do not necessarily hold the same worth for local interpreters, drivers, and team members as for the researcher. Such discrepancies magnify when research is arranged in advance, across countries. Although a valuable connection and friendship had been developed with an experienced and culturally sensitive interpreter over the first two visits to Mongolia, the interpreter found a permanent position just before the commencement  of fieldwork and could not be part of the research team. Good interpreters are in demand and do not necessarily feel loyalty to a specific research project; understandably, as research projects only offer employment for a limited time period. Furthermore, as described by Bujra [55], highly skilled interpreters are expensive and may not be budgeted for. As our research project had a tight schedule (both in terms of finances and season), the selection of interpreters was, unfortunately, a rather rushed process. In retrospect, more time should have been set aside for finding and training the most suitable interpreter. Here, Bujra’s *Lost in Translation? The Use of Interpreters in Fieldwork* [55] offers practical advice including the implications of researching through a third party, negotiating relationships, and choosing interpreters. Anticipating the difficult tightrope that researchers must sometimes walk during this type of research, enables the researcher to plan for these moments. Ethnobiologists should allocate enough time for learning how to deal with the unexpected, despite and within limits of research schedules and financial resources.

Many ethnobotanists strongly encourage the inclusion of local research partners and community participation [14, 24]. In their ethnoveterinary interview guide, Grandin and Young [28] highlight the need for cultural sensitivity, but also caution researchers against assuming that local interviewers (extension agents, etc.), by virtue of being local, automatically have the required knowledge, language skills, and cultural sensitivities for researching local knowledge systems. We must avoid such automatic confidence in new research team members of even though the locals may believe in their own linguistic and research abilities and project such competence. Our fieldwork experience reflected this misalignment, as local members of the research team did not always have the necessary cultural understanding, balance of power, and objectivity during interviews.

Insensitive or power-loaded behavior by drivers, interpreters, or research assistants can offset the interview in irreparable ways. Researchers should, in selecting and working with assistants in general, remember that, to them, it is just one job among others, and they do not necessarily share the same passion and commitment to the research that the researcher has. At the same time, assistants need some basic human qualities and cultural sensitivity (besides honesty) to do the job, as much as researchers need to.

Building rapport with respondents is an essential part of effective interviews and interactions and includes the following research behaviors: a shared understanding of lived experience (in this case, the first author participating in the Mongol Derby and coming from a farming and livestock background); empathy with people’s circumstances; enthusiasm and passion for the work; and knowledge of behaving in culturally appropriate ways (for example, the first author, although being a vegetarian, eating meat during fieldwork, and offering culturally appropriate gifts [60]). Notably, empathy is a basic prerequisite for any engaged fieldwork as it is an engagement skill that opens up an understanding of multiple perspectives. Without a capacity and effort to see things from culturally and personally distant perspectives, and to be respectful of these, it is not possible to carry out good fieldwork.

### Power dynamics

Central to TEK research, including ethnoveterinary research (as experienced through this study), is that a researcher must understand the context in which local or traditional knowledge is located [61, 62]. As pointed out by Etkin et al. [63], there is a need for researchers to recognize and acknowledge that local knowledge is embedded in a much larger system and influenced by many factors, including social, political, and economic circumstances. For our research, participating in and completing the Mongol Derby endurance race represented an opportunity for experiential learning. Strength-related endurance activities such as horse riding, wrestling, and archery are central to Mongolian society. Furthermore, as horse riding is highly respected by Mongolians and is open to women, partaking in an endurance event such as the Derby enabled the first author to earn and gain the respect of local Mongolians, from herders to academics. In addition, riding Mongolian horses and overnighting with herder families during the race, introduced and deepened the lead author’s interest in Mongolia, and allowed her to gain crucial insight into the daily lives of Mongolian herders, and the feasibility planning for a research project. An endurance event of this type can also build mental strength, tenacity, and resilience, essential characteristics for doing fieldwork in places and situations beyond one’s comfort zone.

Despite contrary advice and concerns, travelling on horseback (especially in accompaniment of a trustworthy local guide) was one of the most important and successful approaches used during fieldwork as it allowed for a more participatory research mode. Although choosing to use horses as a means of transport came with some challenges, such as a decrease in the daily distance covered and challenges around sourcing suitable horses and gear, it soon became clear that travelling on horseback was the more appropriate mode of transport in this equine-intensive study environment (elsewhere, adopting local bicycles, canoes, etc. might work similarly). Respondents seemed to feel more at ease and the common topic of horses acted as an “ice-breaker” leading to naturally stimulated conversation around livestock care. In addition, travelling on horseback allowed the primary researcher to use a mode of transport that she felt very comfortable with and allowed for a more etic or culturally experiential understanding of the herder way of life.

Together with an understanding of the local context, a researcher should be aware of the influence and dynamics of positions of power. It is important to think about who holds what power during fieldwork, whether it is during interviews, informal discussions, or the planning of logistics, between researchers and respondents, as well as researchers and local team members, but also between interpreters (often from the city) and respondents (often from more rural areas). While a researcher’s self-assessment may be one of vulnerability in a new place and culture and dependent on local participation, to locals, researchers are powerful in a number of ways, especially when research is undertaken by a Westerner researching non-Western knowledge systems, and where the entire research process and related benefits lies more with the researchers than the "researched" [6]. For our fieldwork, we chose a number of methods to somewhat balance the positions of power between researcher and respondents. Arriving at interviews on horseback rather than in a four-wheel drive vehicle was one such measure, as were participating in culturally important activities (such as eating meat despite the primary researcher being a vegetarian for over 20 years); beginning discussions about ethnoveterinary medicine with a free listing opportunity; and emphasizing the researcher’s role of being a learner in the process. However, despite these methods and approaches, we must acknowledge that the researcher, as a foreigner with limited language ability, experience, and time in the local context, and having certain (known and unknown) preconceived ideas, certain imbalances of power were inevitably present. If nothing else, we hope, that through our interactions, local Mongolian herders became aware that, in contrast to any outsiders who disrespect their culture, other outsiders recognize their wealth of knowledge and the importance and power thereof, as we demonstrated.

Although academic research institutions play a major role in fieldwork related to a research project, it is important to also connect with local boundary organizations. Boundary organizations are those that bridge the gap between research and practice, or science and management, and facilitate the working together and building of relationships between scientists and non-scientists [47, 48]. Although researchers should, ideally, immerse themselves in the local culture and live or engage with the community over a long-term period, the practical reality is that grant holders, funders, and university schedules do not and cannot always take preparation time into consideration. In these cases, boundary organizations can form a link between scientific research and community engagement and have experience with local customs and culture, provide important support for fieldwork. In our experience, the genuine interest and support received from an international development agency boundary organization located in Ulaanbaatar helped immensely, especially in terms of interpreter support, finding suitable horses, field sites, and other fieldwork logistics.

### Gender relations

Within the larger field of TEK research, researchers’ likely adjustments and responses to local norms around gender context, influence, and related issues are often overlooked (until problems arise) [64]. Although fieldwork usually has a particular focus and specific research outputs and requirements, fieldwork that involves people and knowledge is influenced by the entire social system [20].

In medicinal plant research, fieldwork data can easily be misinterpreted due to not being viewed within the context of gender roles within the specific research context, creating potential tension between researcher/interpreter and respondents [65]. In our study, we interviewed both men (*n* = 24) and women (*n* = 26), and the research team consisted of men and women. This balance, together with the experience of working with both female and male interpreters, allowed for a better understanding of gender-related knowledge and differences in the particular Mongolian context. In designing and implementing more inclusive, gender-neutral research, Pfeiffer and Butz [64] suggest using a mixed-gender team as this lessens the likelihood of experiencing cultural restrictions in data gathering. However, as noted by Logan and Huntley [66], the potential impact of gender power dynamics within the research team itself must be taken into consideration and includes possible gender-based translation issues that can arise if culturally induced power relations influence the interview [67].

As in many pastoralist societies, women in Mongolian herding families have many roles and duties to fulfil, from preparing daily meals and looking after children, to processing medicinal plants and seeing to sick livestock. In consideration of this context, interviews with women were mostly held inside the *ger*, (to allow them to continue with chores, such as cooking and preparing food), while interviews with men were often conducted outside the *ger* (Fig. 3).

During interviews, participants were asked whether they perceive any differences in medicinal plant knowledge held by men and women. The majority of respondents (70%, *n* = 29) indicated no difference, illustrated by comments such as “*What is important, is the individual’s interest in medicinal plants”*, “*there is a good exchange between men and women”* and “*there is no difference, it depends on the person’s interest.*” Six respondents (15%) perceived men to be more knowledgeable about medicinal plants, and equally, 15% (*n* = 6) of respondents perceived women to be more knowledgeable, with one respondent mentioning that men know more about choosing plants and women know more about the preparation thereof [34]. Howard [33] remarks that across the world post-harvest processing and preservation are roles often assigned to women and girls. From our study, reasons for men knowing more about medicinal plants included “*men know more because they go with the animals,”* “*it’s easier for men to travel to the mountains where medicinal plants grow,*” and “*men know more because they are closer to the animals.*” Explanations for women being more knowledgeable about medicinal plants included “*women know better than men, men just fly on their horses without seeing what plants are under them,”* and women being more sensitive than men. During interviews where both husband and wife were present, sometimes the wife would take the lead and sometimes the husband, depending on the question. In two interviews however, the wives seemed to be more knowledgeable, but would only share information when asked directly by their husbands. As livestock herding is primarily the work of men and young children, men generally have wider access to grazing lands, forests, hard to access mountainous areas, and other sacred sites than do women. However, women have direct involvement with the use of medicinal plants as they are responsible for sick animals. Women often treat animals close to the home, especially in winter where herds are kept near the homestead. In addition, because most medicinal plants are dried and stored for later use, women are directly involved with the processing and preparation of ethnoveterinary medicinal plants. Although the 26 interviews with women together with participant observations and informal discussions gave valuable insight into gender roles and ethnoveterinary medicinal knowledge held by women, very few (*n* = 2) interviews were held with only women present (respondent and research team). It is, therefore, possible that respondents omitted sensitive topics or certain ailments, and that Mongolian herder women’s full wealth of knowledge was not recorded. In addition, it is possible that, as a foreigner, the primary researcher was not aware of all cultural sensitivities regarding gender. For example, did all women feel comfortable enough to discuss castration or fertility-related livestock problems in the presence of a male interpreter and horse guide? Although it can be assumed that there are fewer sensitive areas around livestock health than human health, and considering that many female-related livestock ailments were openly shared by men and women, it is for the reasons and uncertainties mentioned above that we refrained from further analyzing the ethnoveterinary data gathered in terms of gender. As noted by Shackeroff and Campbell [6], it is important to understand why people will or will not share information, and that gender roles, expectations and taboos, as with any cultural realm, are dynamic and constantly changing due to both internal and external influences.

Gender complexities between a young female researcher, male research assistants, and older male respondents from a mostly patriarchal society are complex and can put female researchers in vulnerable situations. Examples from our fieldwork experience include having to override decisions made by a very assertive male driver, working through the consequent conflict, and dealing with alcohol-related problems and the uneasiness and misperceptions around these difficulties. As a foreign woman doing research in a male-dominant society, one has to prepare to diplomatically handle sensitive situations, such as an interviews being disrupted owing to alcohol-related reasons.

From the first author’s experience, having a partner as a research assistant and introducing him as a husband greatly helped in dealing with and reducing gender-related vulnerabilities. As also experienced by Congdon [46], the presence of a husband (or a wedding band) can provide security and credibility, prevent untoward advances or remarks, and offer much needed support in dealing with gender- and culture-related tensions. Acknowledging and addressing vulnerabilities related to gender, alcohol, and general safety, as well as familiarizing oneself with the necessary skills and tools to deal with these challenges are vital, especially for young female scientists doing research in foreign countries. We advise single female researchers working in highly gendered or patriarchal societies, regardless of their own ideals of female autonomy, to either bring a research assistant or support person with them, or, if that is not feasible, consider hiring or offering co-researcher status (and co-authorship) to a local male or elder woman [68, 69]. In addition, we suggest that novice female researchers connect with more experienced female researchers in similar fields [70], or study the fieldwork accounts and reflections of other female researchers such as those offered by Congdon [46] and Momsen [71].

### Loneliness and researcher well-being

Although research ethics and manuals focus on the well-being of research participants (and correctly so), not much is written about the well-being of the researcher [67]. However, it is necessary that researchers are aware of the potential mental and emotional hardships of fieldwork and the need to protect their well-being accordingly [44]. As described by Hewlett [18], “adapting to the field is a process of learning and overcoming not simply the loneliness that comes from being away from friends and family, but the *aloneness* and shock of being a stranger in an unfamiliar world of bewildering languages, beliefs, and customs.” Pre-empting and planning for research strain and fieldwork weariness are essential in ensuring the researcher’s health and well-being, not only for the researcher, but also for the research’s validity and the correct interpretation of research findings [45].

Peculiar dissonant, challenging emotions can surface during foreign fieldwork. Doing research far from home comforts and support blends the psychic clamor of managing new work tasks, constant socializing with strangers, and feelings of loneliness and estrangement from belonging. Pressures from the drive for data collection and proving academic competence [72] together with limits on time and finances make self-care seem extravagant and postponable, which eventually and predictably leads to exhaustion. This impedes one’s ability to remain flexible and adaptable, which encumbers the cardinal social research goal of building good rapport. In addition, participant observation as part of the research process requires a willingness and commitment to engage in the social worlds in which fieldwork is being done, which can often be physically and emotionally draining [73].

Physical and psychological support mechanisms are crucial for the well-being of a researcher [44] and should include enough time for advanced preparedness, support from fellow researchers, opportunities for reflection, and access to therapy in different forms [45]. From the primary researcher’s experience, having someone on the fieldwork team that understood her background was essential for psychological well-being, continued enthusiasm and motivation for the project and for effective and successful fieldwork. In addition, help from a psychologist in addressing post-traumatic fieldwork-related stress played a critical role in writing up and completing the research dissertation.

Our experience with a research team of an ethnobotanist, a botanist, and an ecologist added valuable input and perspective from various fields. In retrospect, the addition of an anthropologist and a veterinarian (for ethnoveterinary studies) could have further supported the pooling of knowledge and resources and preparation for fieldwork challenges. Researchers should allow time for such collaborations and write it into proposals. Furthermore, it is crucial that grant holders, funders, and academic institutions are aware that this type of social-scientific research requires enough time and funding, and that they offer the necessary support and understanding [74].

Indigenous knowledge researchers should receive social and more specifically, anthropological training in the skills and tools required for establishing genuine rapport with respondents, conducting culturally sensitive interviews, dealing with non-research-related crises, and in ensuring researcher health and well-being during and after the research period [44]. The challenges of fieldwork may not end in the field. Time-off from fieldwork to regain strength and motivation, and to process the difficulties of interacting with a different culture in an unfamiliar social background, should form part of research planning. In addition, post-fieldwork reintegration and associated difficulties should be planned for or, at least, acknowledged. Both formal and informal opportunities for debriefing should be in place so researchers can discuss personal responses to fieldwork and get and give support [45].

In many fields of study, there has been a tendency to not talk or write professionally about fieldwork stress, regrets, disappointments, or the emotional consequences of long-term fieldwork [20, 45], which can be detrimental to other, especially novice, researchers [75]. This need for transparent and reflexive fieldwork accounts is highlighted by the descriptions of fieldwork problems by researchers such as Hallowell et al. [39] and contributors to Hewlett’s [18] volume that can provide vital support and should, therefore, form part of recommended pre-fieldwork reading, to which we hope to contribute here.

## Conclusion

Although the use of ethnobotanical manuals and the theory behind certain methods is of critical importance, it should be emphasized that prescribed and suggested methods need to be adapted to the specific context in which field research is to take place. Researchers should expect to adjust and calibrate their data gathering process according to the human context and the dynamic shifts of culture and politics within the research area. Within this context, we offer a critical and constructive review of the methods used for an ethnoveterinary study in Mongolia, in the hope that our reflections on these methods, complications, and associated recommendations will aid long-distance ethnobotanists with some forethought and pre-emptive management strategies for such hurdles.

While one general aim of TEK research is to collect detailed and quantifiable data, there is a need to both recognize and emphasize that the collection of these data is done primarily through qualitative methods [6]. These include social interactions such as interviews, participant observation, and intensive engagement with knowledge holders. It is these crucial social interactions together with the required ethical conduct, sensitivity, empathy, experience, and skill that researchers should prioritize. In addition, it is important that researchers set aside ample time and provide for the development of meaningful relationships with local team members and respondents, and maintaining researcher well-being. Just as academics understand financial support as an essential resource for a research project, they must likewise value intangibles, including supporting and acknowledging passion, resilience, time, resourcefulness, and flexibility. We suggest that researchers prioritize the need to remain flexible and adaptable, as well as to expect the unexpected, while remaining sensitive yet resilient.

Our review offers an experiential account of several fieldwork and method difficulties that researchers inevitably experience if they are unprepared for such challenges, new to the field, and intimidated to change tactics accordingly. Most long-term ethnobiologists know of intelligent, motivated junior-researchers who have dropped out of programs or changed career trajectories when such difficulties strike. Furthermore, because such people often quit, or sweep novice hardships "under the rug" later, there is a relative dearth of literature guiding new researchers on the types of challenges to anticipate and overcome. While anthropologists generally receive methods training on the ethnographic skills that ethnobiological field-workers need, a considerable proportion of ethnobiologists do not come from anthropology programs.

Therefore, we strongly encourage researchers to reflect on, write about, and publish their experiences in the field as these reflections are crucial for understanding data and results, and can help other researchers improve fieldwork preparations and conduct. By providing a framing grounded in empirical evidence, our reflections point to a range of “softer” skills and awareness needed that might help others navigate a similar pathway. Furthermore, a review of this type is useful for researchers in related fields, mostly for pedagogical purposes, but for grant writing and publishing too.

## Data Availability

The datasets used during the current study are available from the corresponding author on reasonable request.
